# A comparison of the clinical characteristics of Chinese patients with recurrent major depressive disorder with and without dysthymia^☆^

**DOI:** 10.1016/j.jad.2011.06.051

**Published:** 2011-12

**Authors:** Wenhua Sang, Yihan Li, Liang Su, Fuzhong Yang, Wenyuan Wu, Xiaofang Shang, Guanghua Zhang, Jianhua Shen, Mengmeng Sun, Liyang Guo, Zheng Li, Lijuan Yan, Bo Zhang, Gang Wang, Guo Liu, Tiebang Liu, Jinbei Zhang, Yanfang Wang, Bin Yu, Jiyang Pan, Yi Li, Chunmei Hu, Lijun Yang, Yongjin Huang, Shoufu Xie, Xueyi Wang, Jiannin Liu, Luxian Lv, Yunchun Chen, Lina Zhang, Yamei Dang, Shenxun Shi, Yiping Chen, Kenneth S. Kendler, Jonathan Flint, Keqing Li

**Affiliations:** a Hebei Mental Health Center, No. 572 Dongfeng Road, Baoding, Hebei 071000, PR China; b Wellcome Trust Centre for Human Genetics, Oxford OX3 7BN, UK; c Fudan University affiliated Huashan Hospital, No. 12 Wulumuqi Zhong Road, Shanghai 200040, PR China; d Shanghai Jiao Tong University School of Medicine affiliated Shanghai Mental Health Centre, No. 600 Wan Ping Nan Road, Shanghai 200030, PR China; e Shanghai Tongji University affiliated Tongji Hospital, No. 389 Xinchun Road, Shanghai 200065, PR China; f Nanjing Brain Hospital, No. 264 Guangzhou Road, Nanjing, Jiang Su 210029, PR China; g No. 4 Affiliated Hospital of Jiangsu University, No. 246 Nan Men Da Street, Zhenjiang, Jiang Su 212001, PR China; h Tianjin Anding Hospital, No. 39 Wu Jia Yao Street, Hexi District, Tianjin 300074, PR China; i Shandong Mental Health Center, No. 49 East Wenhua Road, Jinan, Shan Dong 250014, PR China; j No. 1 Hospital of Medical College of Xian Jiaotong University, No. 277 West Yan Ta Road, Xian, Shaan Xi 710061, PR China; k No. 1 Hospital of Zhengzhou University, No. 1 East Jianshe Road, Zhengzhou, He Nan 450052, PR China; l No. 1 Mental Health Center Affiliated Harbin Medical University, No. 1 Wu Rui Jie, Nangang District, Haerbin, Heilongjiang, PR China; m Mental Health Center of West China Hospital of Sichuan University, No. 28 Dian Xin Nan Jie, Wu Hou District, Chengdu, Si Chuan 610041, PR China; n Beijing Anding Hospital, Capital Medical University, No. 5 An Kang Hutong Deshengmen wai, Xicheng District, Beijing 100088, PR China; o ShengJing Hospital of China Medical University, No. 36 Sanhao Street, Heping District Shenyang, Liaoning 100004, PR China; p Shenzhen Kangning Hospital, No. 1080, Cui Zu Street, Luo Hu, Shenzhen 518020, PR China; q No. 3 Affiliated Hospital of Zhongshan University, No. 600 Tian He Road, Tian He District, Guangzhou, Guangdong 510630, PR China; r No. 1 Hospital of Shanxi Medical University, No. 85 Jiefang South Road, Taiyuan, Shanxi, PR China; s Mental Hospital of Jiangxi Province, No. 43 Shangfang Road, Nanchang, Jiangxi 330029, PR China; t The First Affiliated Hospital of Jinan University, No. 613 Huang Pu Da Dao Xi, Tian He District, Guangzhou 510630, PR China; u Wuhan Mental Health Center, No. 70, You Yi Road, Wuhan 430022, PR China; v No. 3 Hospital of Heilongjiang Province, No. 135 Jiao Tong Lu, Beian, Heilongjiang, PR China; w Jilin Brain Hospital, No. 98 Zhong Yang Xi Lu, Siping, Jilin 136000, PR China; x The First Hospital of China Medical University, No. 155 Nanjing Bei Jie, He Ping District, Shenyang 110001, PR China; y Dalian No. 7 People's Hospital & Dalian Mental Health Center, No. 179 Ling Shui Lu, Gan Jing Zi District, Dalian, PR China; z The First Hospital of Hebei Medical University, No. 89 Donggang Road, Shijiazhuang 050031, PR China; aa Lanzhou University Second Hospital, Second Clinical Medical College of Lanzhou University, No. 82, Cui Ying Men, Lanzhou, Gansu Province 730030, PR China; ab Psychiatric Hospital of Henan Province, No. 388 Jian She Zhong Lu, Xin Xiang, Henan, PR China; ac The Fourth Military Medical University affiliated Xijing Hospital, No. 17, Changle West Road, Xi'an, Shaanxi 710032, PR China; ad No. 4 People's Hospital of Liaocheng, No. 47 Hua Yuan Bei Road, Liaocheng, Shandong 252000, PR China; ae Guangzhou Brain Hospital/Guangzhou Psychiatric Hospital, No. 36 Ming Xin Lu, Fang Cun Da Dao, Li Wan District, Guangzhou 510370, PR China; af Clinical Trial Service Unit, Richard Doll Building, Old Road Campus, Roosevelt Drive, Oxford, OX3 7LF, UK; ag Virginia Commonwealth University, Department of Psychiatry, Virginia Institute for Psychiatric and Behavioral Genetics, Richmond, VA 23298-0126, USA

**Keywords:** Major depressive disorder, Dysthymia, Symptom, Comorbidity

## Abstract

**Background:**

The relationship between major depressive disorder (MDD) and dysthymia, a form of chronic depression, is complex. The two conditions are highly comorbid and it is unclear whether they are two separate disease entities. We investigated the extent to which patients with dysthymia superimposed on major depression can be distinguished from those with recurrent MDD.

**Methods:**

We examined the clinical features in 1970 Han Chinese women with MDD (DSM-IV) between 30 and 60 years of age across China. Logistic regression was used to determine the association between clinical features of MDD and dysthymia and between dysthymia and disorders comorbid with major depression.

**Results:**

The 354 cases with dysthymia had more severe MDD than those without, with more episodes of MDD and greater co-morbidity for anxiety disorders. Patients with dysthymia had higher neuroticism scores and were more likely to have a family history of MDD. They were also more likely to have suffered serious life events.

**Limitations:**

Results were obtained in a clinically ascertained sample of Chinese women and may not generalize to community-acquired samples or to other populations. It is not possible to determine whether the associations represent causal relationships.

**Conclusions:**

The additional diagnosis of dysthymia in Chinese women with recurrent MDD defines a meaningful and potentially important subtype. We conclude that in some circumstances it is possible to distinguish double depression from recurrent MDD.

## 1 Introduction

Major depressive disorder (MDD) and dysthymia are two common psychiatric disorders where the clinical presentation is typically dominated by a dysphoric mood. While MDD is typically episodic, and durations can be as short as two weeks, dysthymia is a form of chronic depression characterized by depressed mood more days than not, and symptoms that last two years or more. Dysthymic patients tend to have a fluctuating course (Akiskal et al., 1980) and often have a superimposed episode of MDD, a phenomenon called double depression (DD) (Keller and Shapiro, 1982). Dysthymia affects approximately 3% of the adult population (Weissman et al., 1988) and has a prevalence of about 36% of outpatients in mental health settings (Markowitz et al., 1992).

The relationship between dysthymia and MDD is complex. The two conditions are highly comorbid (Markowitz et al., 1992; Weissman et al., 1988) and it remains unclear whether the two conditions reflect two separate disease entities or different aspects of a single condition (Coryell et al., 1994; Keller et al., 1995; Spalletta et al., 1996). Some distinctions have been made: dysthymia and DD differ from MDD in that they have poorer outcomes in naturalistic follow-up studies (Klein et al., 2000), greater Axes I and II comorbidity (Markowitz et al., 1992) and are more likely to have a family history of mood and personality disorders (Klein et al., 1988, 1995; Riso et al., 1996). Few studies have systematically examined, in European populations, differences in patients with recurrent MDD without a history of dysthymia and DD.

In this report, we examine a large sample of carefully assessed Han Chinese women with recurrent major depression, to determine if the clinical course and pattern of comorbidity differ in those with and without a history of dysthymia. To our knowledge, this is the first time these questions have been addressed in data from an East Asian population. Compared to women with recurrent MDD without a history of dysthymia, we expected DD patients to exhibit a higher level of depressive symptomatology, an earlier onset of depression, higher rates of familial aggregation and greater comorbidity especially with anxiety disorders (Kasch and Klein, 1996; Weissman et al., 1984).

## 2 Methods

### 2.1 Subjects

Data for the present study draws upon the ongoing China, Oxford and VCU Experimental Research on Genetic Epidemiology (CONVERGE) study of MDD. These analyses were based on a total of 1970 cases recruited from 53 provincial mental health centers and psychiatric departments of general medical hospitals in 41 cities in 19 provinces and four central cities: Beijing, Shanghai, Tianjin and Chongqing and 2597 controls who were recruited from patients undergoing minor surgical procedures at general hospitals or from local community centers. All cases and controls were female and had four Han Chinese grandparents. Cases and controls were excluded if they had a pre-existing history of bipolar disorder, any type of psychosis or mental retardation. Cases were aged between 30 and 60, had two or more episodes of MDD, with the first episode occurring between 14 and 50 and had not abused drug or alcohol before the first episode of MDD. Controls were chosen to match the region of origin of cases, were aged between 40 and 60, had never experienced an episode of MDD and were not blood relatives of cases. An older minimal age of controls was used to reduce the chances that they might have a subsequent first onset of MDD. The mean age (and SD) of cases and controls in the dataset was respectively 45.1 (8.8) and 47.7 (5.5).

All subjects were interviewed using a computerized assessment system, which lasted on average two hours for a case and one hour for a control. All interviewers were trained by the CONVERGE team for a minimum of one week in the use of the interview. The interview includes assessment of psychopathology, demographic and personal characteristics, and psychosocial functioning. Interviews were tape-recorded and a proportion of them were listened to by the trained editors who provided feedback on the quality of the interviews. The study protocol was approved centrally by the Ethical Review Board of Oxford University and the ethics committee in participating hospitals in China.

### 2.2 Measures

The diagnoses of depressive (Dysthymia and Major Depressive Disorder) and anxiety disorders (Generalized Anxiety Disorder, Panic Disorder with or without Agoraphobia) were established with the Composite International Diagnostic Interview (CIDI) (WHO lifetime version 2.1; Chinese version), which classifies diagnoses according to the Diagnostic and Statistical Manual of Mental Disorders (DSM-IV) criteria (American Psychiatric Association, 1994).

The interview was originally translated into Mandarin by a team of psychiatrists in Shanghai Mental Health Centre with the translation reviewed and modified by members of the CONVERGE team. Phobias, divided into five subtypes (animal, situational, social and blood-injury, and agoraphobia) were diagnosed using an adaptation of DSM-III criteria requiring one or more unreasonable fears, including fears of different animals, social phobia and agoraphobia that objectively interfered with the respondent's life. The section on the assessment of phobias was translated by the CONVERGE team from the interview used in the Virginia Adult Twin Study of Psychiatric and Substance Use Disorders (VATSPUD) (Kendler and Prescott, 2006).

Additional information was collected using instruments employed from VATSPSUD, translated and reviewed for accuracy by members of the CONVERGE team. The history of lifetime major depression in the parents and siblings was assessed using the Family History Research Diagnostic criteria (Endicott et al., 1975). The stressful life events section, also developed for the VATSPSUD study, assessed 16 traumatic lifetime events and the age at their occurrence. Neuroticism was measured with the 23-item Eysenck Personality Questionnaire (Eysenck and Eysenck, 1975), which was also an established instrument for measuring neuroticism.

Both the case and control interviews were fully computerized into a bilingual system of Mandarin and English developed in house in Oxford, and called SysQ. Skip patterns were built into SysQ. Interviews were administered by trained interviewers and entered offline in real time onto SysQ, which was installed in the laptops. Once an interview was completed, a backup file containing all the previously entered interview data could be generated with database compatible format. The backup file, together with an audio recording of the entire interview, was uploaded to a designated server currently maintained in Beijing by a service provider. All the uploaded files in the Beijing server were then transferred to an Oxford server quarterly.

### 2.3 Statistical analysis

Statistical analyses were performed using the software package SPSS 17.0 (SPSS Inc., Chicago, IL). We performed logistic regression analyses to estimate the association of dysthymia with MDD and comorbid disorders. Linear and logistic regression models were used to determine the association of between measures. Coefficient values, odds ratios and 95% confidence intervals were used to quantify the strength of associations. The statistical significance for all tests was set at P < 0.05 and corrected where necessary for multiple testing using a Bonferroni correction.

## 3 Results

### 3.1 Sample characteristics

From our total of 1970 cases with recurrent MDD, 354 met criteria for dysthymia. Comparing those with and without a diagnosis of dysthymia, we found no significant difference in age at interview, marital status or level of education. We then examined the two groups for differences in the onset and course of MDD and other potentially important risk factors, especially personality and family history.

As seen in Table 1, patients with dysthymia had a significantly earlier age of onset of MDD, more depressive episodes and a significantly longer “longest episode” compared to MDD patients without a history of dysthymia. Dysthmic patients had significantly higher levels of neuroticism, reported a significantly greater number of stressful life events and were likely to have one or more first-degree family members with a history of MDD.

### 3.2 Comorbidity

We compared lifetime prevalence rates of 6 anxiety disorders in MDD patients with and without dysthymia (Table 2). MDD patients with a history of dysthymia have significantly higher rates of panic disorder, GAD and agoraphobia, social and blood injury phobias, and animal and situational phobias (the P-value for animal phobias remains significant after applying a Bonferroni correction for testing nine measures (corrected threshold is P = 0.0056)). We also observed significantly higher rates of post-natal depression in the dysthymic patients. However there was no significant difference in the rates of melancholia between the two groups.

## 4 Discussion

Our study addresses an issue concerning the nosology of mood disorders in a large carefully characterized cohort of Han Chinese women with major depression. We investigated the extent to which patients with recurrent MDD with dysthymia could be distinguished from those with recurrent MDD without comorbid dysthymia. We uncovered a number of important differences between these two groups, suggesting that the additional diagnosis of dysthymia in cases of recurrent MDD defines a meaningful and potentially important subtype.

Our major findings are that the cases with dysthymia have a more severe MDD, as indexed by both the number and length of episodes. They had greater co-morbidity with anxiety disorders, higher neuroticism scores and a stronger familial loading for MDD. They had experienced greater environmental adversity as indicated by the number of reported serious life events.

These results are broadly consistent with what has been seen in Western studies contrasting MDD patients with and without dysthymia and showing the former to have greater comorbidity (Markowitz et al., 1992) and increased rates of a family history of MDD (Klein et al., 1988, 1995; Riso et al., 1996).

How can we explain the differences we observe between our cases of recurrent MDD with and without dysthymia? The distinguishing features are a mixture that does not easily fall into neat categories. The greater severity of illness and higher familial/genetic loading might suggest a more biological disorder (Kasch and Klein, 1996; Weissman et al., 1984). However, no excess rates of melancholia were seen in our DD cases, arguing against a more “biological/endogenous” picture. Furthermore, the presence of dysthymia was associated with evidence for greater environmental adversity. The picture of high levels of neuroticism and high comorbidity with anxiety disorders could be interpreted as a more “neurotic” depressive picture, consistent with the origin of dysthymia in DSM-III with the alternative title of “depressive neurosis.” Further studies will be needed to examine more closely the etiologic relationship between MDD cases with and without a history of dysthymia.

Our study benefits from considering the literature on cross-culture variation in dysthymia. A series of large community-based studies conducted in several countries by the World Health Organization shows substantially higher estimates for dysthymia in high-income countries than in low and middle-income countries (Kessler and Ustun, 2008). Furthermore, the same studies reveal variation in severity: whereas 21% of patients were rated as “severe” in Germany, 46% of patients in China and 59% of patients in Nigeria were so rated. However, wherever it occurs dysthymia confers a substantial risk of suicide (Nock et al., 2009) and considerable impairment in functioning (Kessler and Ustun, 2008).

Part of the cross cultural variation in dysthymia can be attributed to the different way depressive symptoms are presented to clinicians, for instance in China somatizing psychological stress via constructs such as neurasthenia (Kleinman, 2004). However it should be noted that, as Phillips et al. (2009) argue, diagnostic rates will vary if more flexible, but still rigorous, structured interviews are used. For example, in a survey of 12% of the adult population of China, only 15 of 16,577 individuals who completed the Structured Clinical Interview for Diagnostic and Statistical Manual (DSM)-IV axis I disorders met research criteria for neurasthenia, while dysthymia emerged as the second most common specific mood disorder (Phillips et al., 2009).

Our results should be considered in the context of several limitations. First, all of the patients were from in-patient hospital samples so that our findings are not representative of community samples (which are likely to include less severely ill patients). Second, our data were collected retrospectively and recall bias will have affected results. Finally, all patients were female, so the applicability of our results to male patients is unknown.

## Role of funding source

Funding for this study was provided by the Wellcome Trust; the Wellcome Trust had no further role in study design; in the collection, analysis and interpretation of data; in the writing of the report; and in the decision to submit the paper for publication.

## Conflict of interest

All authors declare they have no conflicts of interest including any financial, personal or other relationships with other people or organizations within three years of beginning the work submitted that could inappropriately influence, or be perceived to influence, their work.

## Figures and Tables

**Table 1:** Relationship between six clinical features of major depressive disorder and dysthymia.

Clinical feature of MDD	P-value	OR	95% CI
Age of onset	< 0.0001	0.96	0.94–0.97
Duration of longest episode	< 0.0001	1.04	1.01–1.05
Number of episodes of MD	< 0.0001	1.03	1.02–1.03
SLE (number of episodes)	< 0.0001	1.24	1.16–1.33
Family history of MD	0.0002	1.27	1.12–1.45
Neuroticism	< 0.0001	1.12	1.09–1.15

MDD: Major depressive disorder. SLE: Stressful life events. OR: Odds ratio. 95% CI: 95% Confidence intervals.

**Table 2:** The relationship between dysthymia and nine disorders comorbid with major depressive disorder.

Comorbid disorder	P-value	OR	95% CI
Agoraphobia	< 0.0001	1.48	1.28–1.70
Social phobia	< 0.0001	1.46	1.28–1.67
Animal phobia	0.0027	1.14	1.05–1.24
Situational phobia	< 0.0001	1.36	1.23–1.51
Blood phobia	< 0.0001	1.32	1.17–1.48
GAD	< 0.0001	1.87	1.47–2.38
Melancholia	0.64	0.93	0.69–1.25
Postnatal depression	< 0.0001	2.05	1.56–2.70
Panic	< 0.0001	2.71	1.96–3.74

GAD: Generalized anxiety disorder. OR: Odds ratio. 95% CI: 95% Confidence intervals.
